# Supplementary figures and images for: Gene signature for prognosis in comparison of pancreatic cancer patients with diabetes and non-diabetes
Source: PeerJ. 2020 Nov 11;8:e10297. doi: 10.7717/peerj.10297 (PMC7666560; doi:10.7717/peerj.10297)

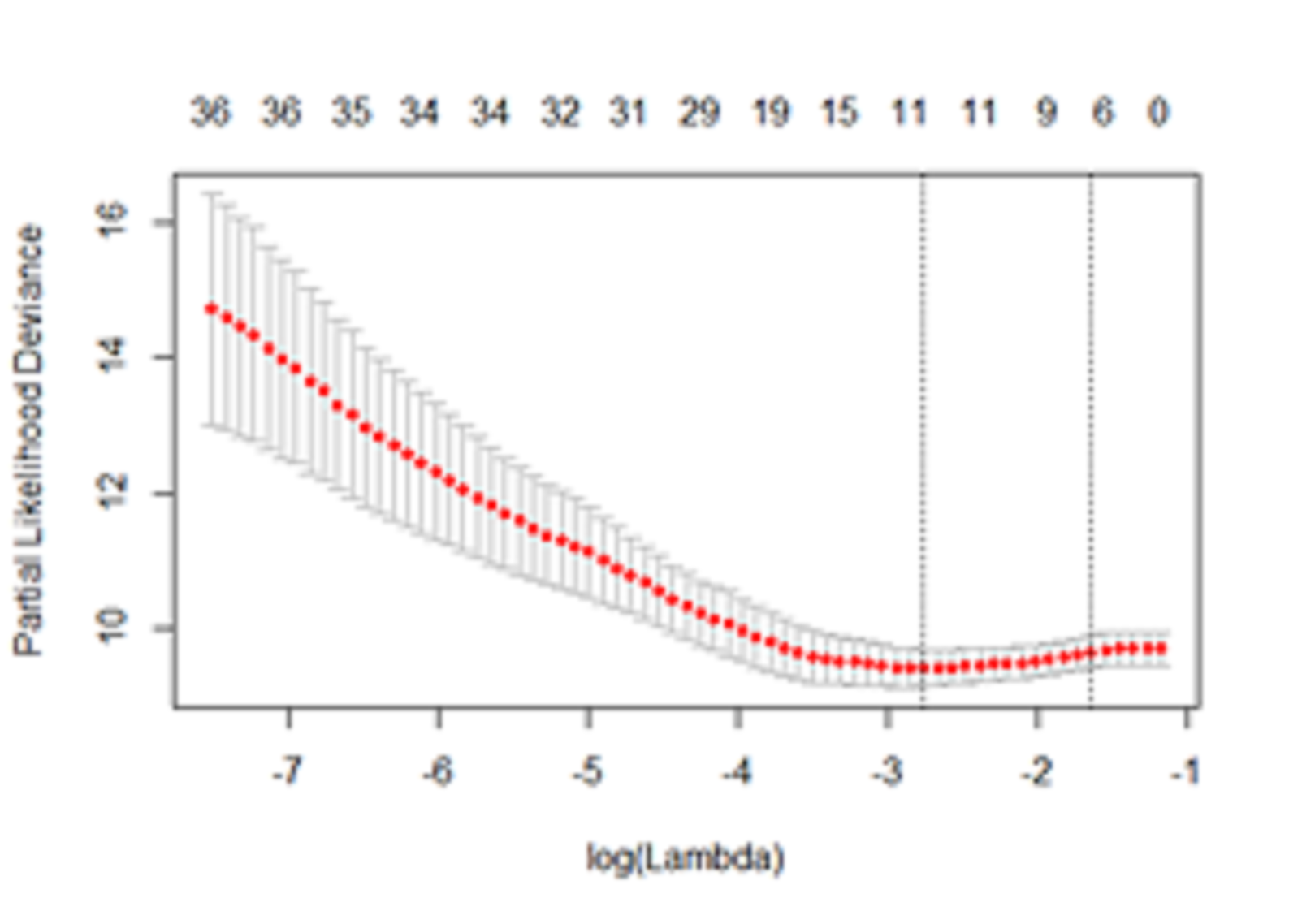

Supplement: Supplemental Information 2 [file peerj-08-10297-s002.png]

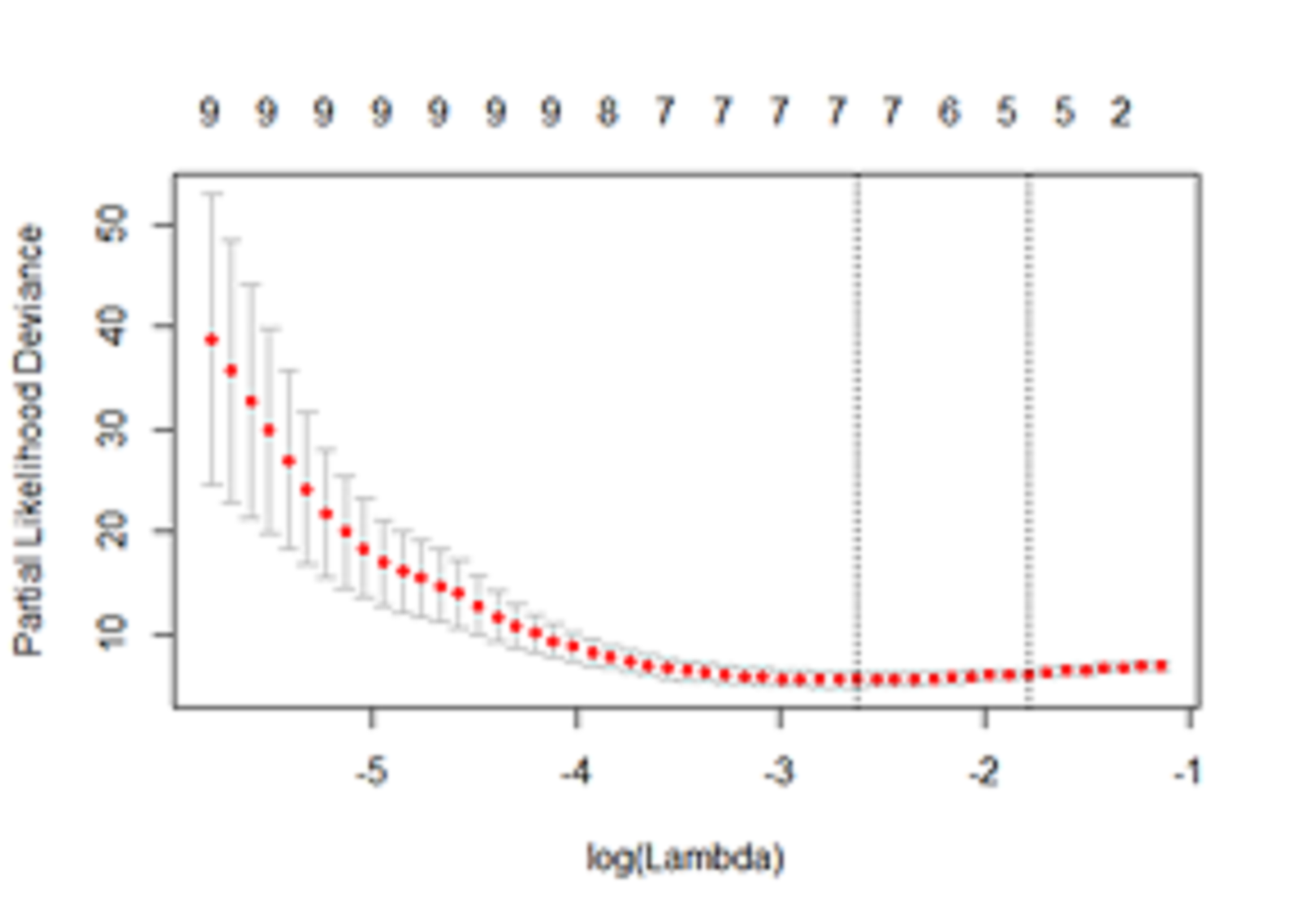

Supplement: Supplemental Information 3 [file peerj-08-10297-s003.png]
